# Solubility of Poorly Soluble Drugs in Phosphatidylcholine-Based Drug Delivery Systems: Comparison of the Loading Capacity in the Bulk Formulation and Its Dispersed State

**DOI:** 10.3390/ph17030400

**Published:** 2024-03-21

**Authors:** Linda Grüne, Heike Bunjes

**Affiliations:** 1 Technische Universität Braunschweig, Institut für Pharmazeutische Technologie und Biopharmazie, Mendelssohnstraße 1, 38106 Braunschweig, Germany; l.gruene@tu-braunschweig.de; 2 Technische Universität Braunschweig, Zentrum für Pharmaverfahrenstechnik, Franz-Liszt-Str. 35a, 38106 Braunschweig, Germany

**Keywords:** phospholipids, self-emulsifying drug delivery systems (SEEDS), phosphatidylcholine-based drug delivery systems, lipid-based drug delivery systems, poorly water-soluble drugs, passive loading, dual centrifugation, charged aerosol detection (CAD)

## Abstract

The aim of this study was to determine the drug loading capacity of phosphatidylcholine-based formulations for four poorly water-soluble drug substances (clofazimine, fenofibrate, artemether, cannabidiol). Two self-dispersing lipid formulations were investigated, which consisted of soybean phospholipids, medium-chain triglycerides and ethanol with a different phospholipid–oil ratio. The direct loading of the bulk formulation was conducted with dual centrifugation, which proved to be a suitable method for screening experiments with the highly viscous formulations. To estimate possible precipitation after dispersion in the gastrointestinal fluids, the solubility of the drugs was investigated in the dispersed formulations. For this purpose, nanodispersions were prepared from the bulk formulations via high pressure homogenization and subsequently subjected to passive loading. A newly developed HPLC method with Charged Aerosol Detection allowed a simultaneous evaluation of the content of soybean lecithin and medium-chain triglycerides in the nanodispersions. When comparing the two phosphatidylcholine-based formulations, a high content of oil was advantageous with regard to a high loading capacity. Drug substances with melting points below 150 °C exhibited a high solubility in the phospholipid-based formulations. A surprisingly high solubility was observed for artemether and cannabidiol with up to 13.0% and 33.3% drug loaded to the formulations, respectively. In the dispersions, a similar solubility as in the bulk formulations was obtained for fenofibrate and cannabidiol. Clofazimine yielded a higher loading result in the nanodispersions than in the bulk formulation.

## 1. Introduction

Many new drug candidates are poorly soluble in water [1,2]. Lipid-based formulations are an interesting approach for the oral delivery of these drugs because they enable the administration of the drug in a dissolved form and can enhance the absorption by forming solubilizing structures in the gastrointestinal tract [3,4,5]. Lipid-based formulations can be simple oily solutions of a drug, but many formulations additionally contain surfactants and co-solvents. These formulations are called self-dispersing or self-emulsifying drug delivery systems (SEEDS) because they disperse in the gastrointestinal tract upon slight movement and contact with the aqueous environment. The fraction of synthetic surfactants and co-solvents in the formulations usually lies between 30 and 60% [5]. In contrast to synthetic surfactants, which can have irritating effects when used in high concentrations [6,7], natural phospholipids are very well tolerated [8,9]. Further, they have emulsifying properties [8] and are therefore an interesting alternative to synthetic surfactants for these formulations. A previously conducted comprehensive miscibility study identified homogeneous and stable mixtures based on phosphatidylcholines as natural emulsifiers. What was developed were both liquid formulations based on oils, and semi-solid formulations based on fats [10]. The most promising results were achieved with mixtures consisting of oils and the soybean phosphatidylcholine Phospholipon 90 G and ethanol as co-solvents. These formulations contained a high fraction of phosphatidylcholine and exhibited self-dispersing properties in simulated gastrointestinal fluids [10]. A subsequent study demonstrated that these formulations can be converted into solid oral dosage forms by filling in hard capsules. Selected formulations exhibited suitable properties for the liquid-filling process and were compatible with hydroxypropyl methylcellulose (HPMC) capsules [11].

Most lipid-based formulations contain the hydrophobic drug in a dissolved state so that the slow dissolution step can be bypassed [12]. The solubility of the drug substance in the drug delivery system must therefore be considered during the development phase. Major challenges with lipid-based systems are, however, the dispensing and mixing of the highly viscous or semi-solid formulations, which are usually necessary for the screening methods used for solubility testing [13]. On a laboratory scale, the loading of lipid-based formulations is often accomplished through the simple magnetic stirring of the formulation until the drug is dissolved. In the case of highly viscous formulations, processing is conducted at higher temperatures [14,15,16]. However, the use of heat is critical when thermo-sensitive drugs and excipients are processed. Elevated temperatures can also lead to the supersaturation of the formulations and, as a result, the delayed precipitation of the drug substance [17,18].

**Figure 1:**
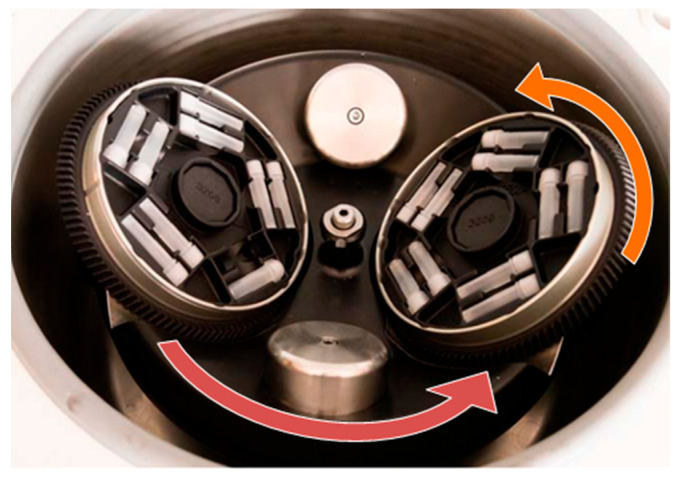
Design of the dual centrifuge ZentriMix with adapters for 2 mL tubes. The rotation around the central axis is indicated in red. Marked in orange is the second axis around which the sample tubes are rotated. Modified from [21].

One method suitable for mixing highly viscous systems is dual centrifugation. Similar to conventional centrifugation, the samples are rotated around a central axis. In addition, however, dual centrifugation involves rotating the sample around a second axis, which causes the directions of the centrifugal forces to change constantly (Figure 1). These superimposed motions result in strong shearing effects and effective homogenization [19]. Dual centrifugation is therefore very well suited for the loading of highly viscous lipid formulations. It is further suitable for laboratory-scale loading screenings [20] as it allows working with small amounts of formulations and enables the simultaneous processing of multiple samples. Since processing is performed in tightly sealed disposable tubes, sterile preparations can be manufactured as well as formulations with toxic or irritating substances [19].

When determining the maximum possible loading capacity of lipid-based formulations, events in the gastrointestinal tract must also be taken into account. After entering the gastrointestinal tract, the formulation will be diluted and later digested. With this comes the risk of the precipitation of the poorly water-soluble drug because the solubility might be lower in the particles formed upon dispersion [12,22]. Therefore, it is also of interest to study the loading capacity of lipid-based formulations in the dispersed state in order to prevent overloading. One possible method is passive loading, which has previously been used in loading studies on lipid carrier systems such as nanoemulsions, nanosuspensions, and colloidal liposome dispersions [23,24]. First, the drug-free carrier particles are prepared, and the drug is then added to the dispersion in powdered form. After sufficient incubation time, the excess drug is removed, and the amount of drug loaded to the dispersion is determined [24]. This method has been successfully used for loading studies with poorly water-soluble drugs and drug candidates [23,24,25,26], needing only very small amounts of the compounds. In this approach, the drug is not thermally stressed. In addition, overloading the carrier particles is unlikely since loading occurs through the diffusion of dissolved drug molecules through the aqueous phase. However, due to the loading mechanism, this approach is not suitable for active ingredients with very low water solubilities of less than 10 ng/mL [27]. An important prerequisite for the determination of the loading capacity in using this method is the reliable separation of the carrier particles from the powdered drug substance.

The aim of this study was to determine the drug loading capacity of phosphatidylcholine-based formulations for different hydrophobic drugs. The direct loading of the bulk formulation was conducted using dual centrifugation. For comparison with the solubility in the dispersed state, nanodispersions were prepared from the bulk formulations and subjected to passive loading, enabling a preview on possible precipitation after dispersion. Furthermore, the influence of the phosphatidylcholine fraction on the loading capacity was evaluated, as well as the impact of the drug loading on the self-dispersing properties of the formulation. A newly developed method was used to simultaneously quantify the phospholipids and oil in the nanodispersion using HPLC with Charged Aerosol Detection.

## 2. Results and Discussion

### 2.1. Direct Loading of the Bulk Formulations

#### 2.1.1. Determination of Solubility through the Detection of Drug Crystals in the Bulk Formulations

**Figure 2:**
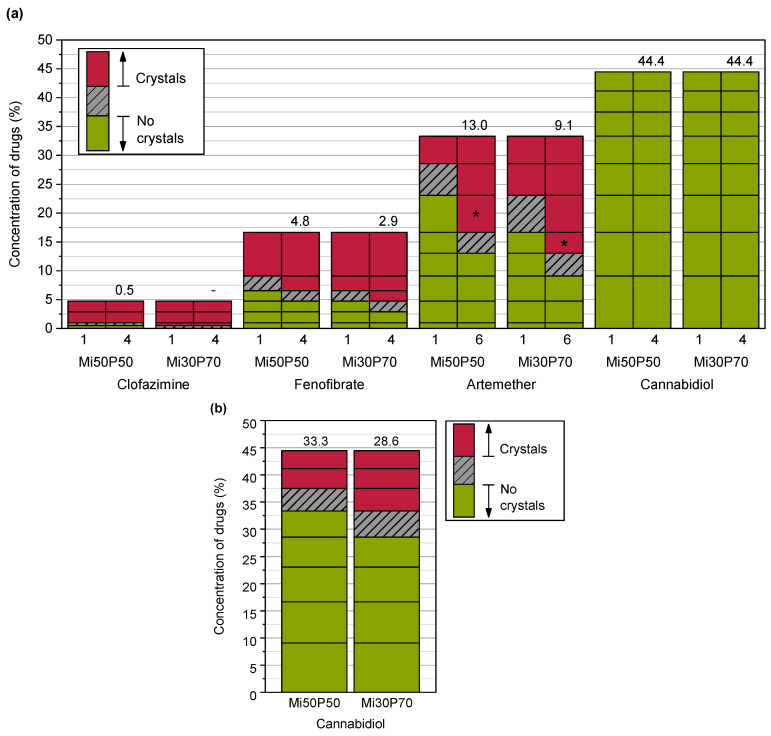
(**a**) Dissolved concentrations of different drugs (%) in Mi50P50 and Mi30P70 as determined through the macroscopic and microscopic detections of drug crystals in the bulk formulation and (**b**) dissolved concentrations (%) of cannabidiol after taking into account the dispersion experiments in phosphate buffer. The formulations were loaded at 23 °C via dual centrifugation. The horizontal lines in the bars indicate which concentrations were prepared. The area above this concentration is shown in red if drug crystals were visible in the bulk formulation (**a**) or pellet (**b**). If no drug crystals were detected, the area below this concentration is shown in green. The numbers above the bars indicate the minimum solubility. The true solubility lies between the investigated concentrations in the grey hatched area. The formulations were examined during a storage period of 18 weeks. For comparison, the results after 1 and 4–6 weeks (numbers below the bars) are shown. No changes were observed after this time. * Repeat tests with artemether showed slightly different minimum solubilities (see text).

The solubilities of poorly water-soluble drugs in bulk phosphatidylcholine-based formulations were determined using dual centrifugation. For these investigations, a formulation with a high (70%) phosphatidylcholine content in medium-chain triglycerides (Miglyol, Mi30P70) was compared to a formulation with a medium (50%) content of phosphatidylcholine (Mi50P50). Dual centrifugation was well suited for screening with these highly viscous mixtures since an effective mixing of the samples was possible both at 23 °C and 65 °C in the heatable centrifuge used in this study. Figure 2 shows the dissolved concentrations (% (*w*/*w*)) of the drug substances in the formulations Mi50P50 and Mi30P70 loaded at 23 °C. The highest concentration obtained without the occurrence of visible drug crystals is called the “minimum solubility” in the following, as the true solubility lies between this concentration and the concentration at which crystals were first visible. When comparing the minimum solubility after 1 and 4 or 6 weeks of storage, it was found that fenofibrate and artemether crystallized with some delay. Although the dual centrifuge can maintain a constant temperature by means of a cooling unit, the high shear forces could have caused a punctual increase in temperature in the sample tubes. This could explain that the supersaturation of the formulations occurred immediately after loading, resulting in delayed precipitation. No changes in the macroscopic or microscopic appearances were observed anymore after 4 and 6 weeks of storage.

**Figure 3:**
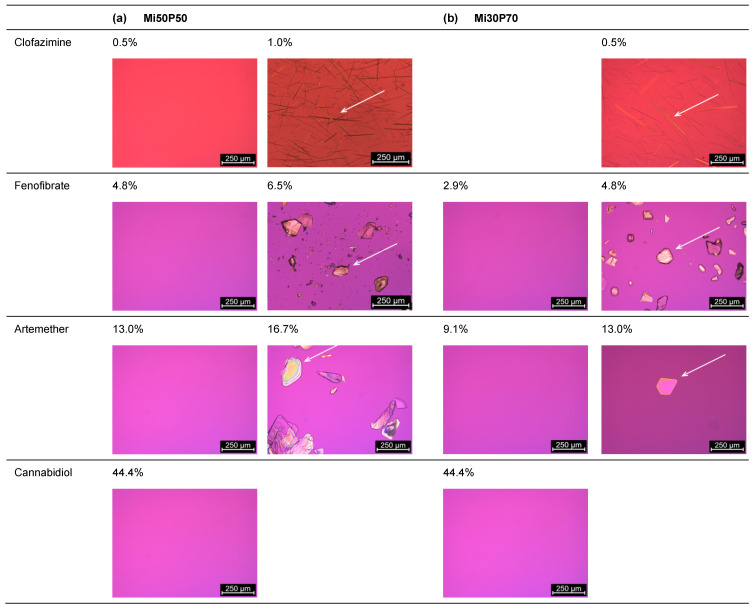
Images recorded through the polarizing microscopy of (**a**) Mi50P50 and (**b**) Mi30P70 loaded with different concentrations of clofazimine, fenofibrate, artemether and cannabidiol. The formulations were loaded via dual centrifugation at 23 °C and stored for at least 4–6 weeks. What is shown are the formulations in which crystals were first visible (**right**) and the last concentration without crystals (**left**). Drug crystals are marked by arrows.

The minimum solubility of clofazimine in the formulation Mi50P50 was determined as 0.5%, since macroscopic and microscopic acicular crystals were observed at 1% drug content (Figure 3). In Mi30P70, acicular crystals were already visible at a concentration of 0.5% clofazimine. Lower concentrations were not examined. For fenofibrate, the minimum solubility in Mi50P50 was 4.8%, and 2.9% in Mi30P70. Accordingly, crystals were visible at drug loads of 6.5% and 4.8% fenofibrate, respectively. In the case of artemether, a repeat test was performed after the first test series for concentrations of 13.0% and 16.7%. For the formulation Mi50P50, crystals were found at 16.7% and higher concentrations in the first trial. In the repeat study, no crystals were visible at this concentration.

In mixture Mi30P70, no crystals were initially found at 13.0% or 16.7%, but in the repeat study, drug crystals occurred at both concentrations. The minimum solubility was set to the lower value of the two series of investigations. Thus, a minimum solubility of 13.0% artemether was determined for Mi50P50 and 9.1% for Mi30P70. Formulations with higher concentrations contained platelet-shaped crystals. In the formulations loaded with cannabidiol, no drug crystals could be observed up to a concentration of 44.4%. Overall, higher drug loads were found in formulation Mi50P50 than in Mi30P70 for clofazimine, fenofibrate and artemether. In the cannabidiol-loaded formulations, such differences were not observed after micro- and macroscopic evaluations.

**Figure 4:**
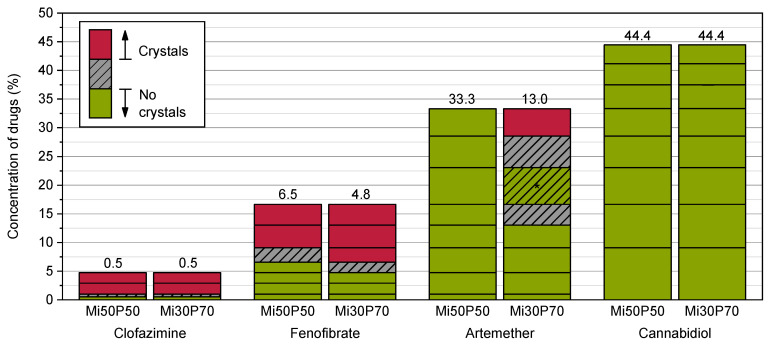
Dissolved concentrations (%) of different drugs in Mi50P50 and Mi30P70 determined by loading at 65 °C. The formulations were checked macroscopically and microscopically for drug crystals in the bulk formulation. The prepared concentrations are indicated by the horizontal lines in the bars. If drug crystals were visible, the area above this concentration is shown in red. If no drug crystals were detected, the area below this concentration is shown in green. The area in between is hatched in grey. Formulations containing clofazimine, fenofibrate and cannabidiol were examined during a storage period of 18 weeks, whereas formulations containing artemether were examined during a storage period of 12 weeks. After 9 weeks, no changes were observed. * For Mi30P70 with artemether, crystals were found in the mixtures with 33.3, 28.6 and 16.7%, whereas no crystals were visible in formulations with a concentration below 13.0% and with 23.1%.

The formulations were additionally loaded at 65 °C to investigate the effect of loading at elevated temperatures, e.g., during the preparation of the mixtures. In contrast to loading at 23 °C, the drugs crystallized over a period of 9 weeks after preparation (Figure 4), after which no changes were seen until the end of the observation period.

The minimum solubility after 9 weeks for clofazimine was 0.5% in Mi50P50 and Mi30P70, and for fenofibrate, 6.5% in Mi50P50 and 4.8% in Mi30P70. Therefore, the minimum solubility when loaded at 65 °C was higher than following the loading process at 23 °C (except for Mi50P50 loaded with clofazimine). Formulation Mi50P50 loaded with up to 33.3% artemether did not show any drug crystals after 9 weeks. With Mi30P70, crystals were found after 9 weeks in the mixtures containing 33.3, 28.6 and 16.7% artemether. In contrast, no crystals were found in formulations with a loading below 13.0% or in the formulation with 23.1%. In formulations loaded with cannabidiol, no drug crystals were visible over the entire observation period, which is consistent with the results of loading at 23 °C.

These results indicate that the formulations were supersaturated with clofazimine, fenofibrate and artemether directly after preparation at 65 °C. It must be assumed that stimuli, such as scratching the wall of the vials, may cause the drugs in these formulations to crystallize. This could be the reason for the irregular results of artemether in Mi30P70. Loading at elevated temperature is often used in the literature to enable the mixing of highly viscous substances [14,15,16]. However, this can lead to the determination of incorrect high loading capacities [2], as the results of this study confirm. With dual centrifugation, the highly viscous formulations could be loaded without using elevated temperatures. This is also an advantage for the processing of thermosensitive substances. It cannot, however, be ruled out that elevated temperatures could occur at certain locations in the sample tubes.

#### 2.1.2. Dispersion of Loaded Bulk Formulations

The formulations were placed in aqueous medium to investigate whether drug crystals precipitated after dispersion. These tests were performed in phosphate buffer pH 6.5 to simulate the pH value in the small intestine. In principle, the solubilities of dissociable drugs could be different in acidic pH in the stomach. Of the active substances investigated, clofazimine is the only dissociable substance. However, at pH 6.5 and pH 1.2, the molecule is mostly positively charged due to its pK_a_ of 9.2. Therefore, a different solubility of this substance is not expected in the stomach and small intestine.

**Figure 5:**
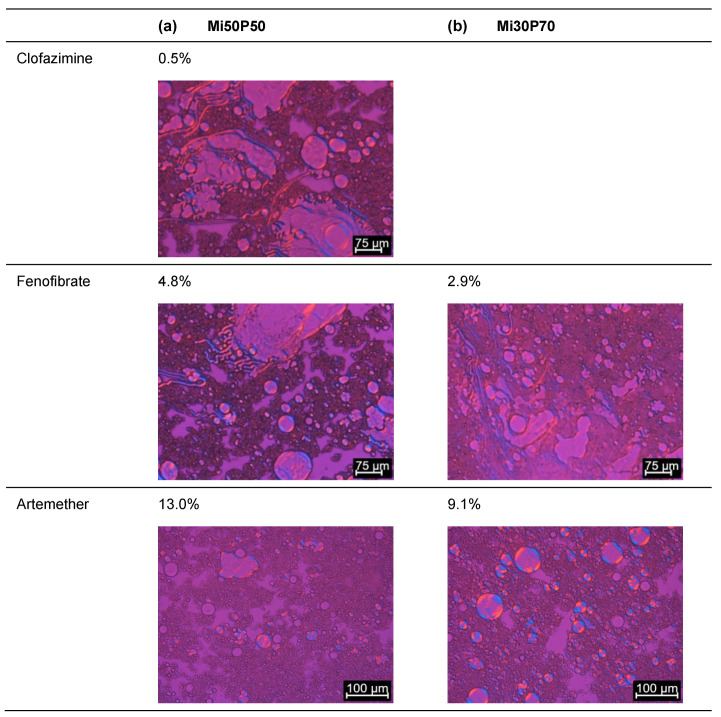

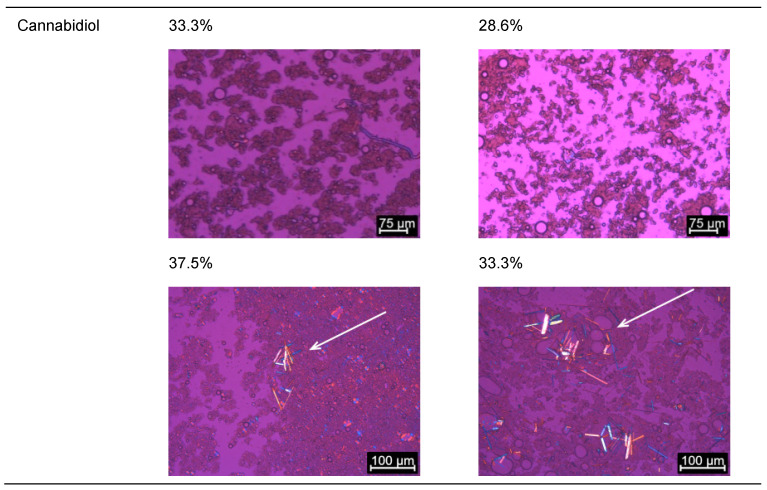
Images recorded through the polarizing microscopy of the pellet of (**a**) Mi50P50 and (**b**) Mi30P70. The loaded formulations were dispersed and centrifuged, and the pellet was examined. In each case, formulations were evaluated in which no drug crystals could be detected in the bulk formulation (loaded at 23 °C). Drug crystals are marked by arrows.

What was examined were mixtures in which no crystals had been detected in the bulk formulation after production at 23 °C. After dispersion and centrifugation, no crystals were found in the pellets of formulations containing clofazimine, fenofibrate or artemether during polarization microscopic examinations (Figure 5). In formulations with cannabidiol, however, drug crystals were observed in the pellets of formulations for which no drug crystals had been detected in the bulk formulations. Taking the dispersion tests into account, the minimum loadable cannabidiol concentration was 33.3% for Mi50P50 and 28.6% for Mi30P70 (Figure 2). The loading capacity, at which the active substance does not precipitate even after dispersion in gastrointestinal fluids, is thus significantly lower for cannabidiol than the attainable loading capacity in the bulk formulation. An explanation could be that after the dispersing of the formulation, the phospholipids are situated at the interface of emulsion droplets and in liposomes. In those particles, the phospholipids might be available for solubilizing the drug substance to a lesser extent than prior to dispersion [28]. In addition, the dissolving capacity of ethanol also decreases due to the strong dilution in the aqueous medium.

Taking into account the loading capacity after dispersion, a higher loading of cannabidiol in the formulation with a higher proportion of Miglyol can be observed in the same manner as for the other drug substances. During storage, the samples containing cannabidiol showed a reddish discoloration that can be traced back to an oxidative reaction of cannabidiol, resulting in colored hydroxyquinones [29]. This reaction occurred even though the formulations were flushed with nitrogen after preparation and loading. It is possible that the oxidation tendency was increased by the presence of the unsaturated fatty acids in the phospholipids. However, a considerable impact on the loading results is not expected, since probably only a small part of the cannabidiol was converted into hydroxyquinones due to the nitrogen treatment.

#### 2.1.3. Evaluation of Loading Capacities

Overall, a higher loading capacity was observed for formulation Mi50P50 compared to Mi30P70 for all investigated compounds. Therefore, a high content of Miglyol seems to be beneficial for drug loading, whereas an addition of phosphatidylcholines reduces the solubilities of the drugs in the formulation. All drugs could be dissolved in the formulations, but the loading capacities were very different. The solubility increased strongly from clofazimine to fenofibrate and artemether to cannabidiol. In the older literature, a logP ≥ 4 was regarded as an indication of good solubility in lipids [28]. The solubility results obtained for the phosphatidylcholine-based formulations show that both artemether (logP 2.8) with its high solubility and clofazimine (logP 7.5) with its low solubility deviate from this expectation. Recent studies suggest that the solubility in lipids depends on factors such as the number of nitrogen atoms, number of double bonds and the size of the polar surface of the drug. A particularly strong influence had the solid-state properties represented by the melting temperature (T_m_) [30,31]. Alskär et al. showed that lipophilic drugs with a melting temperature below 150 °C had comparatively high solubilities in glycerides, whereas only small amounts of drugs with a higher T_m_ dissolved in the lipids [31]. A similar trend was found in investigations on the solubility in colloidal lipid emulsions, in which drugs with a melting point of 147 °C and above showed a low loading in the lipid particles [23].

This tendency could also be observed in the investigations in the current study for the phosphatidylcholine-based formulations. While only small quantities of clofazimine (T_m_: 210–212 °C) dissolved in the formulations, medium to high loading could be achieved with fenofibrate (T_m_: 80.5 °C), artemether (T_m_: 86–88 °C) and cannabidiol (T_m_: 67 °C). Therefore, the results of these loading studies are in line with those of the previous studies by Alskär et al. [31]. Compared to fenofibrate, however, which exhibited the highest solubility of all investigated drugs in medium-chain triglycerides in the study of Alskär et al. [31], the solubilities of artemether and cannabidiol in the phospholipid-based formulations were surprisingly high. These drugs, therefore, seem to be particularly well suited for phospholipid-based formulations. The solubility in the formulations is sufficient to achieve realistic doses of these drugs in a single-dosage form based on doses found in marketed products (20 mg of artemether in Riamet^®^ and 100 mg/mL of cannabidiol in Epidyolex [32]). Further, it was previously shown that the formulations can be liquid-filled in hard capsules and are compatible with HPMC capsules [11].

Loading at 65 °C resulted in the supersaturation of the formulations, which remained stable over the storage period and apparently caused a higher minimum solubility than that when loading at 23 °C. However, dual centrifugation at room temperature probably also caused a certain temperature increase in the individual sample tubes, which temporarily increased the solubility of the drugs. After cooling down to room temperature, the loaded formulations probably became supersaturated, which is why fenofibrate and artemether crystallized with a delay. In the case of artemether, two independent loading series in the range of 13.1–16.7% showed different results with regard to whether the drug crystallized. This can probably be explained by the fact that the formation of a crystallization nucleus is a random event.

When comparing formulations with different phosphatidylcholine contents, it was found that all drugs had a higher solubility in Mi50P50 than in Mi30P70. A high phospholipid content was therefore disadvantageous to the loading capacity of the bulk formulation. This is a difference to many synthetic surfactants, in which drugs often have a higher solubility compared to glycerides. However, these substances often lose their solubilization power when the formulation is dispersed [28,33]. In the phosphatidylcholine-based formulations, this was observed only for cannabidiol, but not for clofazimine, fenofibrate or artemether.

### 2.2. Passive Loading of Dispersed Formulations

#### 2.2.1. Preparation of Nanodispersions

**Figure 6:**
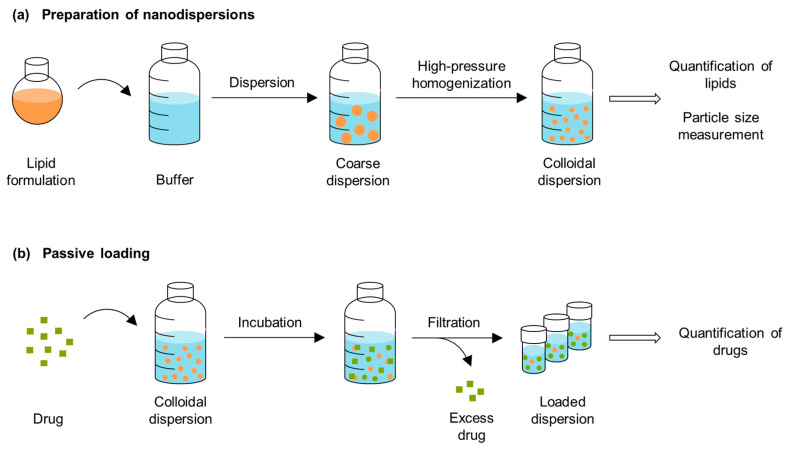
Schematic overview of (**a**) the preparation of the nanodispersions and (**b**) the passive loading procedure.

The drug can precipitate after dispersing the lipid-based bulk formulation in the gastrointestinal tract. Passive loading was evaluated as a method to investigate the loading capacity of the formulations after dispersion. In order to quantify the drug solubilities in the dispersed formulations using this method, it is necessary to separate the undissolved drug from the solubilized fraction. After a simple stirring of the formulations, the dispersions had mean particle sizes of approx. 15 µm (see Section 2.3). The drug could not be separated through the centrifugation of the coarse dispersions because the pellets contained lipid particles in addition to drug crystals (Figure 5). To enable separation through filtration, the particle sizes of the dispersions were reduced using high-pressure homogenization. Figure 6 provides a schematic overview of the preparation of the nanodispersions and the passive loading process.

Processing by means of high-pressure homogenization may lead to changes in the lipid content and composition of the dispersions. During processing, the dispersion can be diluted through process water contained in the dead volume of the microfluidizer or concentrated through the evaporation of water. In addition, coarse particles can be retained by the subsequent filtration step. For this reason, the lipid content of the dispersions was determined to allow an exact calculation of the loading capacity in relation to the lipid components. Furthermore, the lipid composition of the dispersion should not change significantly during processing. This could be caused, for example, by the retention of oil droplets during filtration, which would change the proportion of phosphatidylcholines compared to oil. Such changes would complicate drawing conclusions about the behavior of the formulation. The concentrations of Miglyol and phosphatidylcholine in the nanodispersions were therefore determined via HPLC.

**Figure 7:**
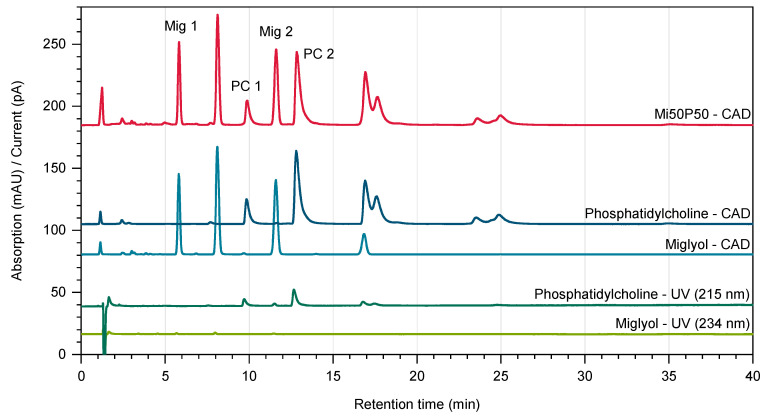
Chromatograms of Miglyol and phosphatidylcholine obtained using Diode Array Detection (UV, shown in green) and Charged Aerosol Detection (CAD, depicted in blue). The chromatogram of a Mi50P50 nanodispersion recorded by the Charged Aerosol Detector is shown in red. For quantification, the characteristic peaks “Mig 1”, “Mig 2”, “PC 1” and “PC 2” were used.

The chromatograms obtained through UV detection displayed only very small peaks for both Miglyol and phosphatidylcholine (Figure 7). In contrast, the chromatograms of the same samples showed significantly larger peaks when detection was performed using Charged Aerosol Detection (CAD), which allowed a reliable quantification. As natural excipients, both Miglyol and phosphatidylcholine are mixtures of different components and were therefore separated into several peaks during the analysis. Two baseline-separated peaks of each material were used for quantification. An analysis of the nanodispersions confirmed that there were no major changes in the proportions of Miglyol and phosphatidylcholine due to high-pressure homogenization (Table 1). The overall lipid contents of the formulations had decreased from 5% to 4.9% for the Mi50P50 nanodispersion and to 4.6% for the Mi30P70 nanodispersion. This indicates a certain dilution of the dispersions through process water.

#### 2.2.2. Stability of Unloaded Nanodispersions

The Mi50P50 nanodispersion exhibited a mean particle size of 151 nm and a PdI of 0.160 after processing. For the Mi30P70 nanodispersion, the mean particle size was 136 nm and the PdI, 0.196 (Table 1). Thus, a higher phosphatidylcholine content in the formulation led to smaller particle sizes after high-pressure homogenization. A closer examination of the particle size distribution curves indicated that the slightly higher PdI of the Mi30P70 nanodispersion is due to a larger proportion of particles below 100 nm, which are presumably unilamellar vesicles.

**Figure 8:**
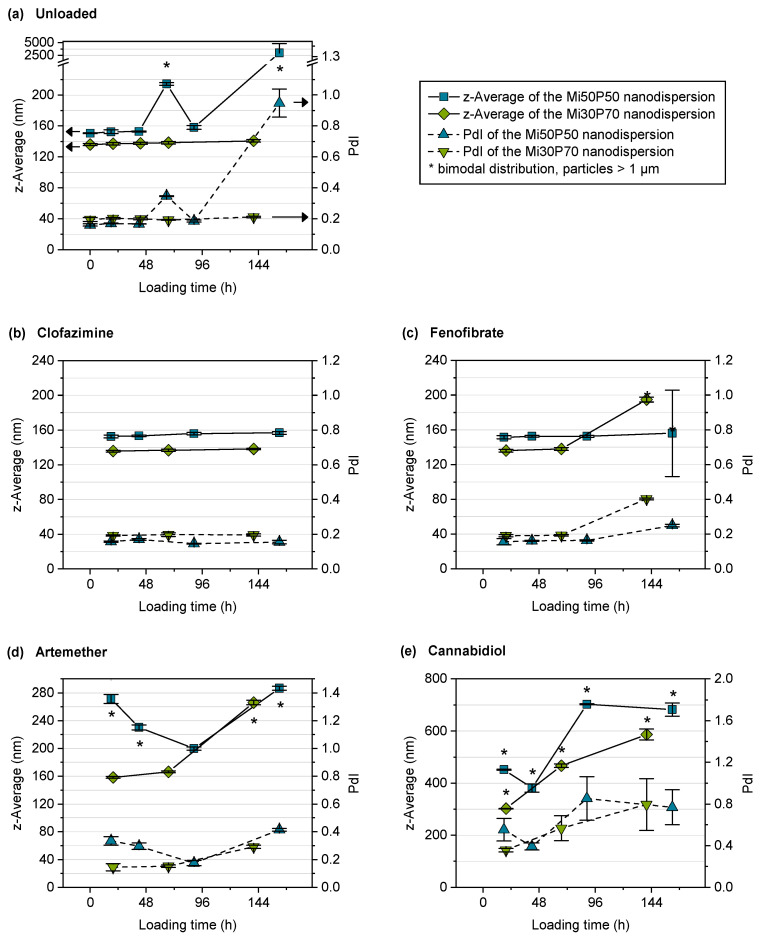
Mean particle sizes (z-average) and polydispersity index (PdI) of the nanodispersions in the course of passive loading. Depicted are (**a**) unloaded control dispersions and dispersions loaded with (**b**) clofazimine, (**c**) fenofibrate, (**d**) artemether and (**e**) cannabidiol (*n* = 3, mean ± standard deviation). Note that the y-axes are scaled differently for the different compounds. Bimodal particle size distributions are marked with asterisks.

Unloaded samples of the dispersions were submitted to shaking for the same time as passive loading was conducted, in order to detect any instability that might occur during loading. The particle sizes of the unloaded dispersions were measured at the respective sampling times of loaded dispersions. After 48 h, the mean particle size and the PdI of the Mi50P50 nanodispersion had increased, and a bimodal distribution was detected (Figure 8a). This indicated an insufficient stability of the dispersion during the loading period. In contrast, the Mi30P70 nanodispersion showed only slight increases in particle size and PdI, indicating a better stability compared to the Mi50P50 nanodispersion. This increased stability correlates with the higher phosphatidylcholine content of Mi30P70.

Phospholipids have emulsifying properties due to their amphiphilic character, as phosphatidylcholine and -ethanolamine accumulate at the interfaces of emulsion droplets and stabilize them by forming a mechanical barrier. However, investigations have shown that pure phosphatidylcholine does not sufficiently stabilize nanoemulsions [34]. In natural lecithin, however, negatively charged free fatty acids and phosphatidic acid are present in small amounts and lead to an additional electrostatic repulsion of the droplets, which greatly improves the stability of the emulsions [34]. Since the phosphatidylcholine used here was highly purified, it can be assumed that it contained only few negatively charged concomitant substances. Therefore, the addition of charged substances such as free fatty acids or the use of less pure phospholipids could potentially enhance the stability of the colloidal dispersions.

Compared to parenteral fat emulsions (e.g., 0.8% phospholipids in Lipofundin MCT 10%, 1.2% phospholipids in Lipofundin MCT 20%, by B Braun [32]), the dispersions investigated here contained a significantly higher content of phospholipids. Due to the high phospholipid content in the formulations, it can be assumed that in addition to emulsion droplets, a high proportion of liposomes was also present in the dispersions [35]. There are indications that the presence of liposomes can destabilize colloidal emulsions [36,37], which could be another explanation for the low stability of nanodispersed Mi50P50. However, this is contradicted by the fact that the Mi30P70 nanodispersion, which contained an even higher proportion of phospholipids and thus presumably also of liposomes, showed a better stability than the Mi50P50 nanodispersion.

#### 2.2.3. Loading of Nanodispersions with Fenofibrate, Artemether and Cannabidiol

**Figure 9:**
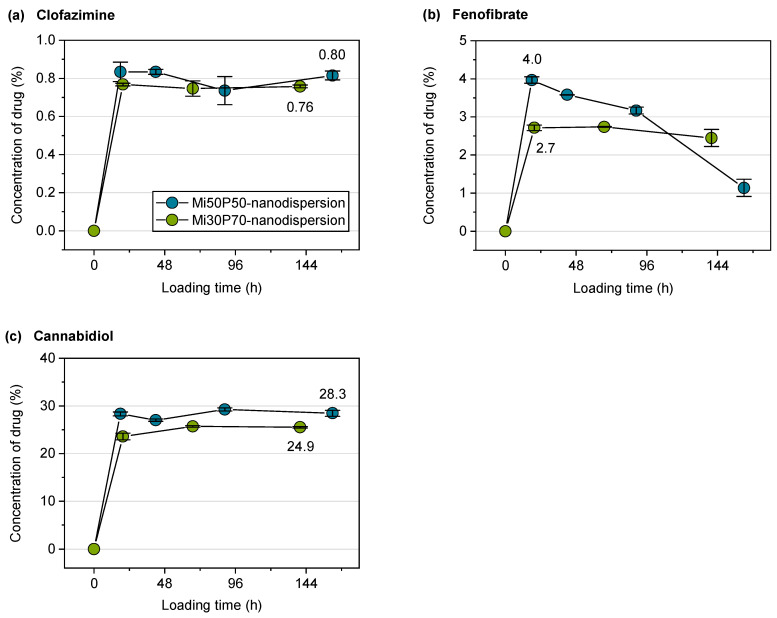
Drug concentrations (%) of (**a**) clofazimine, (**b**) fenofibrate and (**c**) cannabidiol related to the lipid content in the dispersions during passive loading (*n* = 3, mean value ± standard deviation). The concentration of artemether could not be determined UV-metrically due to a weak chromophore and a superposition with the background absorption of the lipids. The mean concentrations of clofazimine and cannabidiol in the plateau are shown at the last measuring point. For fenofibrate, the concentration at the first measuring point is indicated, as there was a strong decrease in the concentration in the course of the experiment.

Despite the limited stability of the Mi50P50 nanodispersion, both dispersions were used for loading experiments. The passive loading process was started by the addition of the powdered drugs to the nanodispersions. At different time points, the excess drug was filtered off, the particle size was measured, and the drug concentration was determined. With respect to the particle size, it should be noted that particles larger than 1 µm might be separated in the filtration step. Nevertheless, the particle sizes of dispersions incubated with fenofibrate, artemether and cannabidiol increased in the course of loading (Figure 8c–e). The results of the drug quantification in the dispersions are shown in Figure 9. It was expected that the drug concentration increases during loading and then reaches a plateau. However, the maximum concentration could also be reached very early, e.g., at the first sampling point.

For the nanodispersions loaded with fenofibrate, the drug concentration measured at the first time point was 4.0% in the Mi50P50 nanodispersion and 2.7% in the Mi30P70 nanodispersion (Figure 9b). In the further course of loading, the concentration of this drug decreased, which indicates that a fraction of loaded particles were retained during filtration. The reason for this would be the formation of larger particles due to the insufficient stability of the dispersion. This is supported by the fact that bimodal particle size distributions were observed at the last sampling time (Figure 8c).

The Mi50P50 nanodispersion loaded with artemether showed bimodal size distributions and an increase in particle size already at the first time point, whereas the Mi30P70 nanodispersion showed this only at the last sampling point (Figure 8d). Therefore, insufficient stability could also be observed for this dispersion. The drug content could not be measured as artemether could not be quantified via UV spectroscopy.

The particle sizes of the cannabidiol-loaded dispersions had already increased at the first sampling point, and the size distribution was bimodal, indicating the destabilization of the nanodispersion (Figure 8e). The drug concentration was, on average, 28.3% in the Mi50P50 nanodispersion and 24.9% in the Mi30P70 nanodispersion and did not further increase at subsequent time points (Figure 9c). Dispersions containing cannabidiol showed a reddish discoloration similar to that of the lipid bulk formulations, indicating the susceptibility of cannabidiol to oxidation.

In the case of fenofibrate and cannabidiol, the concentrations of drugs passively loaded into the nanodispersion was slightly below the concentration achieved by loading the bulk formulation. The increase in the measured particle size during loading indicates that some particles coalesced, therefore indicating the insufficient stabilization of the colloidal particles. It is probable that parts of the drug-containing lipid phase were separated during filtration, which is supported by the decreases in the fenofibrate and cannabidiol concentrations in the course of loading. Therefore, the measured concentrations of the drug probably do not correspond to the true loading capacities in the dispersed state. This must be considered the main limitation of applying the passive loading approach on the presented phosphatidylcholine-based formulations.

The increase in particle size was in some cases more pronounced than in the unloaded Mi50P50 nanodispersion, and it also occurred in the Mi30P70 nanodispersion. In addition, the high loading with cannabidiol resulted in a stronger increase in the measured particle sizes than after the comparatively low loading with fenofibrate. Therefore, it can be assumed that the loading with drug substances destabilized the nanodispersions. According to the literature, the destabilizing effects of some drugs have also been observed in the case of loading phospholipid-stabilized nanoemulsions, leading to coalescence or phase separation. For some drugs, a higher concentration leads to a faster coalescence [37,38]. This effect has also been observed in stability studies on nanoemulsions using a shaking test [37]. All in all, these findings coincide with the results of the present study, which show that loading with drug substances can decrease the stabilities of the dispersions in dependence on the drug concentration.

#### 2.2.4. Loading of Nanodispersions with Clofazimine

No increase in particle size was observed in nanodispersions loaded with clofazimine. However, it cannot be excluded that large particles were separated during filtration and were therefore not detected during particle size measurements. There was no decrease in the concentration of the drug during loading, which would indicate the separation of larger, coalesced particles. This observation supports the assumption that the nanodispersions remained stable in this case. The loading capacity of the Mi50P50 nanodispersions was 0.8% and therefore agrees with the results of the direct loading of the bulk formulation, which revealed a solubility of 0.5–1%. The result of 0.8% for the loading capacity of the Mi30P70 nanodispersion was higher than expected from direct loading, where a solubility below 0.5% was found. The higher loading capacity determined for the dispersion possibly indicates a localization of the drug substance at the interface of the dispersed particles. In loading investigations with nanoemulsions, drugs with high melting points were localized to a large extent at the interface instead of in the core of the particles. Furthermore, they were loaded to a higher extent to liposomes than to emulsions [23]. A reduction in the particle size to the nanometer range generally leads to an enlargement of the interface of emulsions. Furthermore, the incorporation into liposomes is possible only after dispersion. Both factors could explain the higher loading capacity of clofazimine in nanodispersions.

In the course of loading with clofazimine, neither an increase in particle size nor a decrease in drug content was observed. Therefore, nanodispersions loaded with clofazimine were more stable than those loaded with the other drugs. On the one hand, because only a small amount of clofazimine was loaded into the particles, this might not yet have a destabilizing effect on the nanodispersions. On the other hand, the Mi50P50 nanodispersion loaded with clofazimine was more stable than the unloaded dispersion, indicating that clofazimine might have a stabilizing effect. In phospholipid-containing emulsions, a stabilizing effect could be observed with drugs that are located at the interface and have a suitable charge [37].

### 2.3. Testing of Self-Dispersing Properties

**Figure 10:**
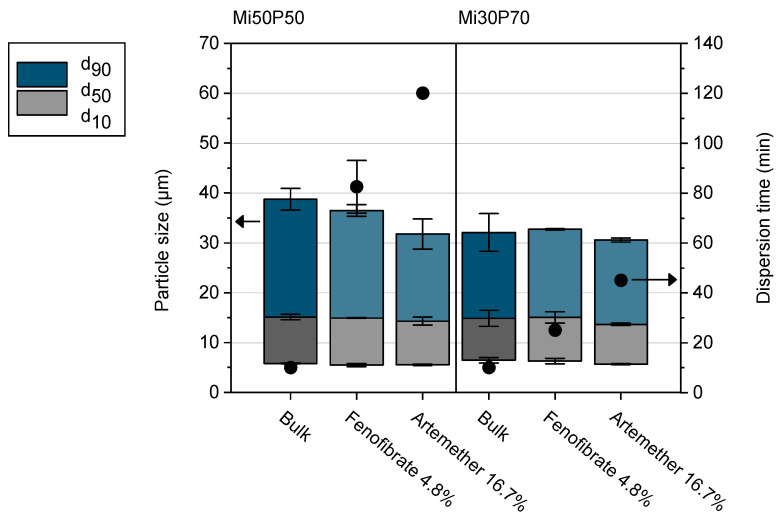
Dispersion time (scatters) and particle size distribution (columns) of selected formulations (*n* = 2–3, mean value ± standard deviation). Formulations loaded with 4.8% fenofibrate or 16.7% artemether are shown in light blue/light gray. The data of the unloaded bulk formulations are shown in dark blue/dark grey for comparison.

In order to investigate how drug loading affects the properties of the formulations, dispersion tests were carried out in Simulated Gastric Fluid (SGF) with selected loaded bulk formulations (Figure 10). For these tests, formulations Mi50P50 and Mi30P70 were loaded with the same concentration of fenofibrate (4.8%) or artemether (16.7%). In most cases, the loaded concentrations were above the minimum solubility, which could lead to the precipitation of drug crystals in the dispersion medium. This phenomenon might change the particle size distribution resulting from dispersion, but an influence on the dispersion time was not expected.

The particle sizes in the dispersions of all loaded formulations were similar to those of the unloaded bulk formulations. Unloaded formulations dispersed in less than 10 min, which was evaluated as suitable for self-dispersing formulations [10]. However, loading with fenofibrate and artemether significantly increased the dispersion time of the mixtures. Formulations with artemether showed higher dispersion times than mixtures with fenofibrate, which can be attributed to the higher drug concentration in the formulations and the resulting reduction in phospholipid content. Furthermore, Mi50P50, which contains a lower amount of phosphatidylcholine, had a higher dispersion time when loaded with the same amount of drug compared to Mi30P70. The presence of the lipophilic drugs therefore had a negative impact on the dispersibility of the formulations, the extent of which depended on the amount of drug introduced and the resulting reduction in phospholipid content, as well as on the composition of the formulation. Therefore, in order to optimize the formulations for loading, a balance must be found between increasing the phospholipid content, which causes better dispersibility, and increasing the Miglyol content, which allows a higher loading.

## 3. Materials and Methods

### 3.1. Materials

The soybean phospholipid Phospholipon 90 G (94–102% phosphatidylcholine, <4% monoacylphosphatidylcholine, tocopherole, ascorbylpalmitate, according to the supplier’s specifications) was a gift from Lipoid GmbH (Ludwigshafen am Rhein, Germany). The medium-chain triglycerides Miglyol 812 (Miglyol) were purchased from Caelo (Hilden, Germany) and ethanol 96% (HPLC quality) was purchased from Fisher Chemical (Loughborough, UK). The model drugs in this study were artemether (Acros Organics, Geel, Belgium), cannabidiol (THC Pharm, Frankfurt am Main, Germany), clofazimine (Acros Organics, Geel, Belgium) and fenofibrate (Cayman Chemical, Ann Arbor, MI, USA).

The Simulated Gastric Fluid (SGF) had a pH of 1.2 and was prepared from hydrochloric acid 37% (Sigma Aldrich, St. Louis, MO, USA) and sodium chloride (Carl Roth, Karlsruhe, Germany) according to the European Pharmacopoeia [39]. Phosphate buffer pH 6.5 contained 10 mmol of sodium dihydrogen phosphate (Merck KGaA, Darmstadt, Germany) and was preserved with 0.05% sodium azide (Carl Roth, Karlsruhe, Germany). Water for the media was purified via bidistillation. For HPLC and for the dilution of samples for photon correlation spectroscopy (PCS), ultrapure water was obtained via filtration and deionization (EASYpure TM LF, Barnstead, MA, USA).

Solvents used for HPLC measurements with Charged Aerosol Detection were of LC-MS quality. Acetonitrile LC-MS and methanol LC-MS were purchased from Carl Roth (Karlsruhe, Germany), and formic acid LC-MS was purchased from VWR International GmbH (Darmstadt, Germany). For HPLC with UV detection, acetonitrile and methanol were used in HPLC-quality from Carl Roth (Karlsruhe, Germany).

### 3.2. Preparation of Lipid Bulk Formulations

Two different formulations were prepared for the loading experiments, which contained 5% ethanol as co-solvent. As it is the same for both formulations, this is not mentioned in the nomenclature of the mixtures. In Mi30P70, the remaining 95% of the formulation consisted of 30% Miglyol (Mi) and 70% phosphatidylcholine (P), and in Mi50P50, it consisted of 50% Miglyol and 50% phosphatidylcholine.

The phospholipids were weighed into 10 mL polypropylene vials (Zscheile & Klinger GmbH, Hamburg, Germany) together with the oil and the final concentration of 5% ethanol. The vessels were gassed with nitrogen, closed with stoppers and crimped. The ZentriMix 380R (dual centrifuge, Andreas Hettich GmbH & Co KG, Tuttlingen, Germany) was equipped with a heating coil to allow processing at elevated temperatures. It was first preheated for 30 min at 65 °C, and the samples were then mixed for 30–60 min at this temperature and 2350 rpm. The formulations were stored at 20 °C–23 °C in a climatic room or climatic chambers (RUMED, Rubarth Apparate GmbH, Laatzen, Germany).

### 3.3. Direct Loading of Lipid Bulk Formulations with Drugs

#### 3.3.1. Preparation of Loaded Formulations

For the direct loading of the bulk formulations, different amounts of the drugs were weighed to a 2 mL screw-cap vial made of polypropylene (Andreas Hettich GmbH & Co KG, Tuttlingen, Germany), and 0.5–1 g of bulk lipid formulation was added. The heatable ZentriMix 380R was preheated for 30 min at 23 °C or 65 °C, and the samples were then mixed for 60 min at 2350 rpm and 23 °C or 65 °C. Afterward, the formulations were gassed with nitrogen and stored at 20 °C in a climatic room. Samples containing cannabidiol were wrapped with aluminum foil to protect them from light.

The concentration of the drug is stated as % (*w*/*w*) of the final formulation. Following percentages were determined for the different drugs: 0.5, 1.0, 2.9 and 4.8% clofazimine; 1.0, 2.9, 4.8, 6.5, 9.1, 13.0 and 16.7% fenofibrate; 1.0, 4.8, 9.1, 13.0, 16.7, 23.1, 28.6 and 33.3% artemether; 9.1, 16.7, 23.1, 28.6, 33.3, 37.5, 41.2 and 44.4% cannabidiol. Separate samples were prepared for each concentration and stored for up to 18 weeks. Replicates were prepared for selected samples with artemether, see Figure 4.

#### 3.3.2. Determination of the Solubility through the Detection of Drug Crystals

The dissolved concentrations (%) of the drugs in the formulations were determined by examining the bulk formulation macroscopically and microscopically for drug crystals. The highest concentration without visible drug crystals was called the “minimum solubility”, as the true solubility lies between this concentration and the concentration at which crystals were first visible. The formulations were checked visually and via polarized light microscopy (Leica DMLM equipped with a Leica MC170 HD camera and Leica LAS X software (version 3.4.2), Leica Microsystems GmbH, Wetzlar, Germany) after 1, 4, 6, 9, 12 and 18 weeks. In addition, it was investigated whether drug crystals precipitated when the formulations were dispersed in simulated gastrointestinal fluids. For this purpose, 50 mg of a loaded sample in which no crystals were detectable in the bulk formulation was dispersed in 10 mL phosphate buffer on a magnetic stirrer (RT 10, IKA-Werke GmbH & Co. KG, Staufen, Germany). After 30 min, the dispersions were centrifuged for 15 min at 10,000× *g* and 23 °C (Allegra 64R, Rotor C1015, Beckman Coulter, Brea, CA, USA), and the pellet was examined for drug crystals via polarization microscopy. Each formulation was dispersed twice, and each pellet was sampled and examined via polarizing microscopy twice.

### 3.4. Passive Loading of the Dispersed Formulations

To investigate the loading of the formulations in the dispersed state, the passive loading method was used based on previously described procedures by Rosenblatt and Bunjes and Göke and Bunjes [23,24]. Initially, aqueous nanodispersions were prepared from the formulations through high-pressure homogenization to enable a reliable separation of the excess drug from the lipid particles through filtration. The nanodispersions were then characterized and loaded. A schematic summary of the entire process is presented in Figure 6.

#### 3.4.1. Preparation of the Nanodispersions

To prepare nanodispersions, 5% (*w*/*w*) of the bulk formulation was predispersed in phosphate buffer by means of a magnetic stirrer and then processed using an Ultra-Turrax (T25 basic, IKA-Werke GmbH & Co. KG, Staufen, Germany) for 5 min at 11,000 rpm. The resulting dispersion was homogenized using a Microfluidizer M-110P (interaction chamber type F12Y DIXC, Microfluidics, Westwood, CA, USA) at 300 bar for 10 cycles (corresponds to approx. 12 min at a batch size of 150 mL). The nanodispersion was then filtered through a 0.45 µm polyvinylidene fluoride syringe filter (Rotalibo, Carl Roth, Karlsruhe, Germany), and the particle sizes were measured.

#### 3.4.2. Particle Size Measurements

The particle sizes of the nanodispersions were measured via photon correlation spectroscopy with a ZetaSizer Nano Series ZS (ZEN3600, Malvern Instruments, Malvern, UK) with 173° backscatter. Before the measurement, the samples were diluted with ultrapure water to a laser attenuation of 6. After an equilibration time of 120 s, 3 measurements of 120 s each were performed at 25 °C. A refractive index of 1.46 was assumed for the dispersed phase, and 1.33 for the continuous phase. To describe the mean particle size and the particle size distribution, the measurements were averaged. The resulting diameter (z-average) and the polydispersity index (PdI) are shown in Table 1.

#### 3.4.3. Passive Loading

To load the nanodispersions, 50 mg of clofazimine, fenofibrate or artemether or 75 mg of cannabidiol were weighed into glass screw-cap vials, and 1 mL of dispersion was added to each vial. The vials were flushed with nitrogen and incubated on a horizontal shaker (Vibrax VXR basic, IKA-Werke GmbH & Co. KG, Staufen, Germany) at 20 °C in a climatic room. Three separate samples were loaded for each time point and combination of drug and nanodispersion. At different time points, the nanodispersions were filtered through a 1 µm glass fiber syringe filter (Carl Roth, Karlsruhe, Germany) to remove excess drug. In addition, drug-free samples of the nanodispersions were shaken as a control, and the particle sizes of these nanodispersions were measured at the respective time points. In addition, the particle size of one loaded nanodispersion each (per combination of drug and nanodispersion) was determined after filtration.

#### 3.4.4. Quantification of Lipids

The following method was developed to quantify the contents of phosphatidylcholine and Miglyol in the nanodispersions via high-performance liquid chromatography (HPLC). A Thermo Scientific Dionex Ultimate 3000 (Thermo Fischer Scientific, Waltham, MA, USA) system with an LPG-3400SD pump and a WPS-3000 TSL autosampler was used for the analysis. A DAD-3000 Diode Array detector as well as a Corona Veo detector employing Charged Aerosol Detection (CAD) and a nitrogen generator (Corona Nitrogen 1010, Peak Scientific Instruments Ltd., Inchinnan, Scotland) were used for detection. Chromeleon Chromatography Data System Software 7.2 was used for instrument control and data evaluation.

An RP18 column (Hypersil Gold, 150 mm × 2.1 mm, particle size 1.9 µm, pore size 175 Å) was connected to a guard column (Hypersil Gold 3UM, 10 mm × 2.1 mm, both Thermo Fisher Scientific, Waltham, MA, USA) and kept at 35 °C using a TCC-3000SD column thermostat. Methanol LC-MS/acetonitrile LC-MS/ultrapure water (53/40/7, (*v*/*v*)) with 0.1% formic acid was used as a mobile phase at a flow rate of 0.3 mL/min. The temperature of the autosampler was set to 10 °C, and the evaporation temperature of the CAD was 35 °C. In total, 200 µL of the nanodispersions were diluted to 10 mL with acetonitrile LC-MS/methanol LC-MS (50:50, *v*/*v*) to fully dissolve the lipids, and 5 µL were injected. Three independent dilutions of each sample were measured. Based on calibration curves for phosphatidylcholine and Miglyol, two baseline-separated peaks of both excipients were used for quantification. The retention times were ~5.7 min (“Mig 1”, R^2^: 0.9942) and ~11.4 min (“Mig 2”, R^2^: 0.9950) for the components of Miglyol, and ~9.7 min (“PC 1”, R^2^: 0.9993) and ~12.6 min (“PC 2”, R^2^: 0.9988) for phosphatidylcholine. The concentrations of the components “Mig 1” and “Mig 2” as well as “PC 1” and “PC 2” were averaged in order to calculate the contents of Miglyol and phosphatidylcholine in the nanodispersions.

#### 3.4.5. Quantification of the Drugs

The drugs were quantified via UV spectrophotometry (Specord 40, Analytik Jena AG, Jena, Germany) using the following self-developed method. 10–30 µL of the loaded nanodispersions were dissolved with acetonitrile/methanol (50:50, *v*/*v*) to 10 mL and measured in a quartz cuvette (Carl Roth, Karlsruhe, Germany). Clofazimine was measured at a wavelength of 280 nm, fenofibrate at 287 nm and cannabidiol at 225 nm. The measured absorbance was corrected for the background absorbance of the dissolved unloaded nanodispersions, which were measured at the corresponding concentration and wavelengths. Calibration lines were established for clofazimine (R^2^: 0.9992), fenofibrate (R^2^: 0.9997) and cannabidiol (R^2^: 0.9996) in the solvent. Artemether could not be determined via UV spectroscopy due to a weak chromophore and an overlay with the background absorption of the dispersion.

### 3.5. Testing of Self-Dispersing Properties

It was tested whether selected mixtures exhibited self-dispersing properties after drug loading. Formulations loaded with fenofibrate or artemether were compared to the unloaded bulk formulation. The tests were conducted using 100 mL of Simulated Gastric Fluid (SGF) in a 250 mL glass vial. The SGF was equilibrated prior to the experiment at 37 °C in climatic chambers (Memmert, Schwabach, Germany). In total, 50 mg of the loaded formulation was weighed onto a small plastic disc, and the disc was added to the fluid to start the experiment. The fluid was stirred at 100 rpm using a magnetic stirring bar and stored in the climatic chamber at 37 °C throughout the experiment. The SGF was visually checked every 5 min as to whether the formulation was completely dispersed. After 30 min, samples were checked every 15 min until the experiment was discontinued after 2 h. Subsequently, measurements of the particle size distributions were conducted through laser diffraction with polarization intensity differential scattering technology (LS 13320; Beckman-Coulter, Krefeld, Germany). After appropriate dilution, 3 runs of 90 s each were performed, and the average values were used to calculate the d_10_, d_50_ and d_90_ values (volume distribution). As refractive indices, 1.46 and 1.33 were assumed for the particles and the aqueous phase, respectively. Two to three experiments were conducted for each formulation.

## 4. Conclusions

Dual centrifugation proved to be well suited for the loading screenings of highly viscous phosphatidylcholine-based formulations. Drugs with melting points below 150 °C (fenofibrate, artemether, cannabidiol) exhibited a medium to very high solubility in these formulations. In particular, cannabidiol and artemether appear to be promising candidates for phosphatidylcholine-based formulations, as their solubilities in the bulk formulations were sufficiently high to achieve realistic doses of these drugs in a single-dosage form.

A high proportion of Miglyol in the formulations had a positive effect on the loading capacity. In contrast, a higher proportion of phosphatidylcholine was more favorable for the dispersibility of the loaded formulations. Therefore, these two aspects need to be balanced during formulation development. The results obtained when loading the nanodispersions indicate a low probability of drug precipitation after the dispersion of the formulations in the gastrointestinal tract, as similar loading results were obtained. Clofazimine yielded a higher loading result in the nanodispersions than in the bulk formulations, whereas similar loading results were obtained for fenofibrate and cannabidiol.

## Figures and Tables

**Table 1:** Characteristics of the nanodispersions of Mi50P50 and Mi30P70 directly after homogenization (*n* = 3, mean value ± standard deviation). The concentrations of Miglyol and phosphatidylcholine were determined via HPLC with Charged Aerosol Detection.

	Mi50P50 Nanodispersion	Mi30P70 Nanodispersion
Fraction of Miglyol in the formulation (%)	49.6 ± 1.4	31.3 ± 0.4
Fraction of phosphatidylcholine in the formulation (%)	50.4 ± 0.9	68.7 ± 0.8
Concentration of lipids in the dispersion (%)	4.9 ± 0.1	4.6 ± 0.1
z-Average diameter of the dispersion (nm)	150.6 ± 0.4	136.1 ± 1.3
Polydispersity index of the dispersion	0.160 ± 0.007	0.196 ± 0.013

## Data Availability

The data are available upon request.
